# Progress in Medicine

**Published:** 1900-06-23

**Authors:** 


### Progress in Medicine.

### RHEUMATISM AND GOUT.

**Treatment.**—Meier[1] notices the value of adding bark or iron to the salicylates in many forms of rheumatism, and when the stomach or head are irritated by that drug he would substitute aspirin, a combination of acetyl and salicylic acid, which has no irritating effects. In the discussion on acute rheumatism and its cardiac complications at the Clinical and Chelsea Societies no general agreement was shown as to treatment. Caton[2] advocated his special plan, when a murmur exists, of absolute rest in bed for several weeks, with a succession of small blisters in the region of the first and the fourth dorsal nerves, and iodides by the mouth. Thus, in 54 cases where the lesion had existed before treatment only 24 were cured, while in 31 where the murmur came on while the patient was laid up no less than 27 were sent home sound. Several speakers emphasised the value of adding alkalies or iodides to the salicylates at a more or less early stage, and agreed that the salicylates and complete rest should be continued for a long while after all fever and pain have been removed. Douglas Powell holds that the diet must be strictly liquid for a considerable time, that water should be allowed freely, and that the patient should be kept in bed for many days after the symptoms have disappeared. Primary endocarditis is generally curable, while mitral stenosis and aortic regurgitation, two of the worst results of rheumatism, only occur at a late stage, and in consequence of a strain continued on a previously damaged valve. Hence the great importance of care in the treatment of the early lesions. G. Sansom[3] refers to the fact that rheumatic endocarditis may occur in the fœtus, and that the evil influence of the disease is very long continued, the toxins either being evolved through many years, or causing such a nerve change that metabolism is chronically prevented. Hence our knowledge of the early stages of heart affections is scanty and vague. The symptoms of pericardial trouble especially vary in the most extraordinary manner, and are of little value in prognosis. As to treatment, he recommends the salicylates and an ice bag in the pericarditis, and defends the practice of adding ammonia to the dose of salicylates, since he finds that no decomposition takes place between them such as some observers have imagined. In the cases of sudden enlargement of the heart, where the symptoms appear and disappear very rapidly, and a condition similar to that of a swollen rheumatic joint probably exists, he would still press the use of salicylates, though at times strychnia and stimulants may be also required. The use of the continuous galvanic current over the vagus area has also shown itself of service in some of these acute cardiac cases. H. Dayton[4] mentions that ordinary salicylate treatment is relied on at the New York Hospital, but that two cases of poisoning by the drug in ordinary doses have occurred there recently. Methyl salicylate is generally applied in addition to the exterior of inflamed joints, and covered over by waterproof tissue. Persistent fluid in the joints is treated with blisters, and if any stiffness remains it is relieved by local hot-air baths. The salicylate is in some cases given by the rectum, or for it is substituted salol or salophen. In sub-acute attacks alkalies and iodides are given with methyl salicylate externally. Bodine,[5] at Mount Sinai Hospital, frequently adds bicarbonate of soda to the salicylate, and recommends that the salicylate be always well diluted and given with water. Favourable accounts of the hot-air treatment continue to appear. E. Satterthwaite[6] shows the importance of keeping the limb in the heating apparatus dry and free from sweat by careful padding. Kessler has treated 350 rheumatic cases with hot-air apparatus. Some had been enabled to return to work which they had not done for years. He gives lactic acid internally, and advises that the hot-air treatment be continued for several months. Manges finds that in acute rheumatism remarkably good results are obtained by rendering the affected joints quite immovable by splints, while a moderate salicylate treatment, combined with sufficient soda to render the urine alkaline, is kept up. He agrees with other writers as to the danger of contracting fresh chills after the hot air bath if the limb be exposed to cold air afterwards. Willis[7] and Dyring report 19 cases treated by the Tallerman method, including rheumatism, lumbago, and sciatica, of which 15 were cured. They find that the younger the patient and the more recent the illness the greater the likelihood of speedy cure. In advanced arthritis deformans of elderly people there is relief from pain after persistence in the treatment, but relapse always follows when it is left off. L. A. Coffin[8] refers to the view that traumatic cases do well under this method, while the results in rheumatic and gouty ones are very unsatisfactory. However, he shows the success with which some apparently rheumatic cases were treated by him, and, though complete cures were not obtained, the joints were made useful once more, and the pain greatly relieved. W. C. Bosanquet[9] states that the cases of acute rheumatism at Charing Cross Hospital amounted in eight years to 450, the months in which most cases occurred being May and November. He notices the high proportion of domestic servants attacked, and the number of cases in which a sore throat was an early symptom (5 per cent. of the whole). Occasionally the disease began by what appeared to be an attack of pneumonia, followed later on by articular troubles. Endocarditis was seen in 137 persons out of the 450, and pericarditis in 28, chorea in 13, erythema nodosum in 9, and subcutaneous nodules in 5. W. J. Morton[10] claims that the high potential, high frequency electric current is the one which gives the best results in chronic rheumatism. The effect of the currents seems to be to increase the tissue metabolism and heat production. Thus, the oxygen absorbed is greater, and the $CO_2$ and urea excreted are augmented, while the patient's general health is immensely benefited. Besides the static induced current and the spark, he employes a form of high potential which he calls the "wave current." He claims that muscular rheumatism, if treated with the spark, ceases to give pain for twelve hours or longer. Repeated sittings cause lengthened intervals and final cure. For chronic rheumatism nothing in the writer's experience equals the spark or one of the other forms of electro-static treatment. He insists that the advance of rhematoid arthritis can be completely arrested by this method, the more quickly if it is in an early stage. A long spark must be applied to each affected joint, and iodide of potassium should be given in increasing doses up to ten grains three times a day. All rheumatic patients are placed by Morton on a meat diet. J. Adler[11] discusses minutely the pathology of muscular rheumatism. It is produced by an interstitial myositis. There is first a local hyperæmia, then an emigration of leucocytes and proliferation of the connective tissues, so that the muscle bundles and fibres are forced apart. Finally absorption or fibrous degeneration may take place, and these processes involve also the neighbouring nerves and joints. Such patches of induration and swelling can be felt in many cases of muscular rheumatism, as the teachers of massage have noticed. The large superficial muscles may be free, but a careful search will often show such a tender patch, not necessarily at the spot where the pain is referred. Adler also declares that mild pyrexia is frequently present, and occasionally even endocarditis. Conversely in persons with cardiac lesions, but no history of articular rheumatism, numerous hard patches may often be felt in the muscles, the remains of former attacks. He finds that rheumatic myositis may thus show traces in various unexpected quarters—in the scalp, in the rectus abdominis, and internal oblique muscles, where it may be mistaken for appendicitis or renal colic. As to treatment, he recommends the salicylates, and especially salol, an ice-bag, and, above all, careful massage after the primary acute stage is over. The entire paper is of great interest as an attempt to lift muscular rheumatism from the uncertainty it is now involved in, "a functional disturbance without structural changes," as it has been called. Adler believes it has an infectious origin, and thinks that the frequent history of sudden strains causing the attacks is due to previously existing tender indurations.

[1] Clinical Excerpts, Feb. [2] Med. Rec., April 7. [3] Lancet, Mar. 31. [4] Med. Rec., April 7. [5] Loc. cit. [6] Loc. cit. [7] Australasian Med. Mag. [8] New York Med. Jour., Mar. 10. [9] Lancet, June 2. [10] Med. Rec., April 21. [11] Med. Rec., Mar. 31.

